# Current Concepts of Metastasis Formation

**DOI:** 10.3390/cancers3032886

**Published:** 2011-07-13

**Authors:** Jörg Haier, Peter Gassmann

**Affiliations:** Comprehensive Cancer Center Muenster, University Hospital Muenster, Albert-Schweitzer-Campus 1, 48149 Muenster, Germany

The development of secondary distant organ and lymph node metastasis has an extraordinary impact on the prognosis of patients with solid cancer. In most cases the advent of metastatic growth represents the turning point from a local, potentially curable, disease to a systemic non-curable situation. As a highly regulated process, metastasis formation follows a distinct, non-random pattern characteristic for each tumor entity. Metastasis formation and strategies to prevent this lethal event in the progression of cancer is of fundamental interest for cancer science and patient care. In this special issue of *Cancers*, papers highlighting cellular mechanisms of metastasis formation, genetic and epigenetic aspects associated with organ and tumor specific metastasis formation, as well as papers outlining experimental and clinical therapeutic concepts for anti-metastatic treatment are included.

Damsky *et al.* [1] suggest to incorporate novel concepts such as metastatic dormancy, premalignant dissemination, and organ-specific metastasis into or models of understanding distant metastasis formation. In their overview they critically summarize initial and recent models regarding the intriguing phenomenon of metastatic dormancy—the time period between removal of the primary tumor and subsequent recurrence of disease. Many processes and phenomena can be attributed to the general process of distant tumor formation. However, specific patterns of metastasis resulted in two concepts of seed and soil [2] and anatomical predisposition [3] for determination of specific organ involvement. The ongoing discussion of the non-exclusive theories is supported by many similarities, but also a large number of specificities during metastasis formation of different tumor types and into different host organs. Beauchesne [4] provides such an example for glioma manifestations.

Tumor microenvironment is of immense importance not only for primary cancers but also for secondary sites [5]. In their review, Wu *et al.* [6] provide a comprehensive review of the role and regulatory interactions of tumor surrounding tissues for the development of lymph node metastasis. These authors critically summarize data that link tumor microenvironment, including inflammation (at the cellular and cytokine levels) and tumor-induced lymphangiogenesis, with nodal metastasis. In a second review dealing with lymph node metastasis formation, Walk and Weed [7] focus on recently identified biomarkers involved in this process. Although they use head and neck squamous cell carcinoma as an example, many proteins described in this review can be considered as general factors for lymph node involvement. One example is the importance of the involvement of chemokines in many if not all carcinoma entities. This group of signaling molecules is one major topic of Thobe *et al.* [8] in their summary of regulators of skeletal metastasis formation.

Understanding the mechanisms of distant metastasis formation has begun to become translated into clinically relevant therapeutic consequences. Chi and Komaki [9] comprehensively discuss the therapeutic options for treating metastases using secondary brain tumors as an example, which arise mostly from a lung cancer primary.
